# PML: Regulation and multifaceted function beyond tumor suppression

**DOI:** 10.1186/s13578-018-0204-8

**Published:** 2018-01-25

**Authors:** Kuo-Sheng Hsu, Hung-Ying Kao

**Affiliations:** 1 Department of Biochemistry, Case Western Reserve University, 10900 Euclid Avenue, Cleveland, OH 44106 USA; 2 The Comprehensive Cancer Center of Case Western Reserve University and University Hospitals of Cleveland, Cleveland, OH 44106 USA; 3 Present Address: Tumor Angiogenesis Section, Mouse Cancer Genetics Program (MCGP), National Cancer Institute (NCI), NIH, Frederick, MD 21702 USA

**Keywords:** PML, Gene expression, Protein modification, Proteolysis, Stem cell and cancer stem cell renewal, Chemotherapy resistance, Metabolism, Inflammatory responses, Neural function, Mammary development, Angiogenesis

## Abstract

Promyelocytic leukemia protein (PML) was originally identified as a fusion partner of retinoic acid receptor alpha in acute promyelocytic leukemia patients with the (15;17) chromosomal translocation, giving rise to PML–RARα and RARα–PML fusion proteins. A body of evidence indicated that PML possesses tumor suppressing activity by regulating apoptosis, cell cycle, senescence and DNA damage responses. PML is enriched in discrete nuclear substructures in mammalian cells with 0.2–1 μm diameter in size, referred to as alternately Kremer bodies, nuclear domain 10, PML oncogenic domains or PML nuclear bodies (NBs). Dysregulation of PML NB formation results in altered transcriptional regulation, protein modification, apoptosis and cellular senescence. In addition to PML NBs, PML is also present in nucleoplasm and cytoplasmic compartments, including the endoplasmic reticulum and mitochondria-associated membranes. The role of PML in tumor suppression has been extensively studied but increasing evidence indicates that PML also plays versatile roles in stem cell renewal, metabolism, inflammatory responses, neural function, mammary development and angiogenesis. In this review, we will briefly describe the known PML regulation and function and include new findings.

## Background

Promyelocytic leukemia protein (PML) was first identified as a fusion partner with retinoic acid receptor alpha resulting from a chromosomal translocation between chromosomes 15 and 17 [1–3]. Since then, evidence has accumulated that PML functions as a tumor suppressor [4, 5]. In mammalian cells, PML is enriched in discrete nuclear substructures referred to PML nuclear bodies (NBs) [6, 7]. In addition to PML NBs, PML is also present in the nucleoplasm and the cytoplasmic compartments, including the endoplasmic reticulum (ER) and mitochondria-associated membranes (MAMs) [8, 9]. It appears that both nuclear and cytoplasmic PML can promote cell apoptosis by distinct mechanisms [8, 10, 11].

As a tumor suppressor, PML protein abundance is frequently low in tumorous tissues [12]. Thus, the regulation of *PML* gene expression, PML protein modification and turnover have been the main subjects of study. For example, PML protein modifications, PML NB formation, abundance and localization are tightly regulated in response to environmental stimuli. Dysregulation of PML and PML NB formation alter PML-associated transcriptional regulation, protein modification, apoptosis and cellular senescence [13].

Due to its initial association with cancer as a tumor suppressor, early studies on PML have mainly focused on its role in apoptosis, cell cycle regulation and tumorigenesis [11]. Nonetheless, recent reports have indicated that PML plays versatile roles in other physiological and pathological settings. These include stem cell and cancer stem cell renewal, drug-resistance, metabolism, inflammatory responses, neural and mammary development and angiogenesis (see below). Together, these findings not only open a new avenue for understanding PML biology but they further highlight the possibility of targeting PML as a potential therapeutic strategy.

## PML protein structure and isoforms

**Fig. 1:**
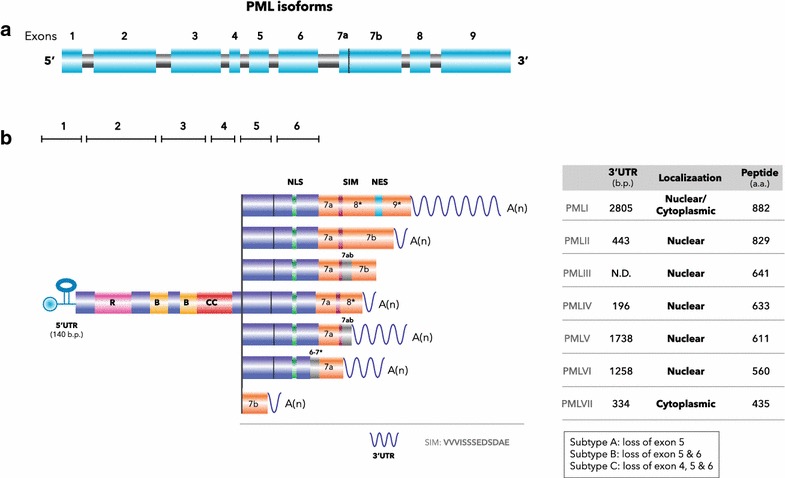
Schematic of PML gene and its isoforms and their localizations. **a** The primary *PML* transcript contains nine exons and eight introns and is alternatively spliced. The exons are shown as blue boxes. **b** The primary *PML* transcript can be alternatively spliced to generate more than 11 isoforms. Only seven *PML* mRNA isoforms that share exons 1–4 are shown. Note that all nuclear isoforms (I–VI) harbor exons 1–6. Isoforms III and V contain introns colored in grey that are spliced out in other nuclear isoforms. The asterisks mark the regions from partial exons or introns. In this Figure, the localization, the molecular weight of PML protein isoforms and the sizes of the *3*′*-UTR* of each *PML* isoform are also shown. *PML* mRNA isoforms harbor unique *3*′*-UTRs*, with a common 140-bp *5*′*-UTR* containing a functional *IRES*. *SIM* SUMO interacting motif, *NLS* nuclear localization sequence, *NES* nuclear exportation sequence)

The PML protein belongs to the family of tripartite motif (TRIM)-containing proteins that consist of more than 70 members in humans characterized by a structurally conserved RING finger/B box/coiled-coil (RBCC) domain [14]. This RBCC motif is preserved among all PML isoforms [15] (Fig. 1). The RBCC domain has been shown to mediate protein-protein interactions and PML NB assembly [16–18]. The nascent *PML* transcript contains 9 exons (Fig. 1a) and can be alternatively spliced into multiple isoforms with variable C-termini. According to classical Jassen nomenclature, PML isoforms can be generally classified into PMLI to PMLVII (Fig. 1b). The nuclear localization sequence (NLS) in exon 6 is not present in the *PML* isoform VII, which is exclusively cytoplasmic [15, 19] (Fig. 1b). The largest isoform, *PML I*, harbors a putative nuclear export signal in the exon 9. Presumably, this isoform is capable of shuttling between nucleus and cytoplasm. In exon 7, a tetrapeptide sequence containing amino acids VVVI is known as a SUMO-interacting motif (SIM), due to its ability to bind sumoylated proteins [20]. Furthermore, a group of sub-class PML variants [19], A, B and C, derived from PML isoform I–VI are documented due to the alternative splicing as shown in Fig. 1b.

## Subcellular distribution of PML

In mammalian cells, 1–30 discrete PML NBs with a size of 0.2–1 μm can be observed in each nucleus [6, 21]. PML is an essential component of PML NBs. To date more than 170 proteins have been found to associate with PML constitutively or transiently [22]. The composition of PML NBs is dynamic and heterogeneous due to the shuttling of PML-associated components and the composition of PML NBs is dictated by specific PML isoforms [7]. It was proposed that sumoylation of PML and SIM-dependent association with sumoylated proteins play a pivotal role in PML NB formation [23]. However, it is clear that sumoylation is not essential for NB formation because a mutant PML (3KR) devoid of sumoylation is still capable of forming NB, though the NB size is generally larger and the number is reduced [24]. PML NB assembly is likely initiated from PML oligomerization, though the residues responsible for the oligomerization remain to be identified [7, 13]. Given that PML-associated components harbor diverse physiological functions, the regulation of PML NBs is key to controlling various biological processes, such as apoptosis, inflammation and angiogenesis (see below).

In addition to localizing to the nucleus, several PML isoforms can be found in the cytoplasm. *PML VII* and *PML I*, as well as other isoforms, like subtype B and C, are potentially cytoplasmic due to the absence of the NLS or the inclusion of the NES (Fig. 1) [8, 19, 25]. Moreover, the truncated PML proteins caused by two distinct pathological missense mutations 1272delAG and IVS3-1 G are localized primarily in the cytoplasm and can sequester nuclear PML, thereby decreasing PML NBs [26]. Interestingly, cytoplasmic PML may possess opposite physiological functions from its nuclear counterpart, depending on the context. The 1272delAG and IVS3-1 G mutant cytoplasmic PMLs show dominant effects over nuclear PML and thereby inhibit p53-mediated transcription and cell growth suppression [26]. In response to HSV-1 infection, cytoplasmic PML is induced by increased alternative splicing, which presumably is part of the strategy employed by viruses to weaken host defenses [27]. A change in cellular redox status also alters cytoplasmic PML levels by redistributing nuclear PML to the cytoplasm. For example, treatment with the antioxidant, sulforaphane in HUVECs results in an accumulation of PML in the cytoplasm [28]. In another example, TGFβ stimulation, cytoplasmic PML or PML VII, exclusively cytoplasmic isoforms recruit Smad2/3 and SARA to potentiate TGFβ signals, cell growth arrest, senescence, and apoptosis [8]. A fraction of PML in mouse embryonic fibroblasts (MEFs) was found localized in MEMs which bridge mitochondria and the ER. Such MEM-associated PML controls calcium flux to the mitochondria by compartmentalizing a large complex that includes PP2A, AKT, and the inositol triphosphate receptor (IP3R) [10]. Given that calcium influx into mitochondria from the ER is a key step in apoptosis, *Pml*^−/−^ MEFs exhibit resistance to ER stress-induced apoptosis. Thus, PML appears to be a critical regulator of apoptosis both in the nucleus and the cytoplasm [10].

## Regulation of PML expression by multiple extracellular stimuli

**Fig. 2:**
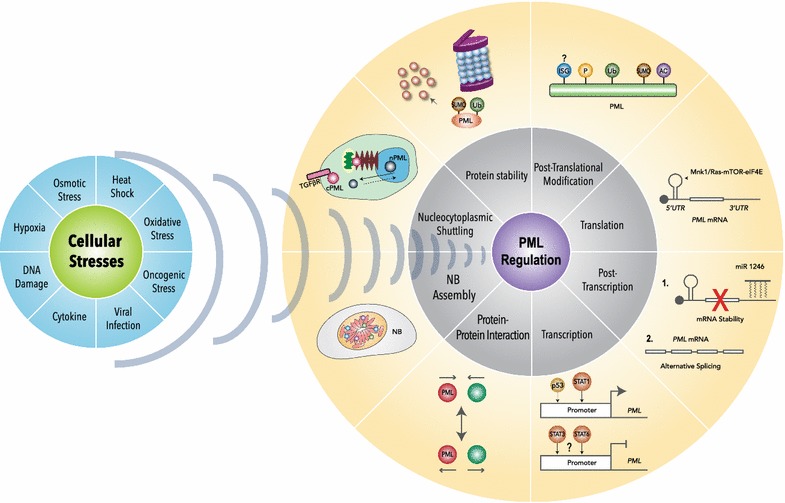
Overview of PML regulation by multiple stimuli and molecular mechanisms. PML is a stress responsive protein. Upon various extracellular stimuli or DNA damage, as shown in the blue circle, PML is regulated by different mechanisms, from transcription, translation to protein-level regulation, shown in the grey and yellow circle. *ISG* isgylation, *P* phosphorylation, *Ub* ubiquitination, *SUMO* sumoylation, *AC* acetylation, *cPML* cytoplasmic PML, *nPML* nuclear PML

PML is a sensor of cellular stress and environmental cues including growth factors and cytokines. The abundance of PML protein is tightly regulated by transcriptional and translational machineries in response to stresses. Additionally, post-translational modification plays a key role in PML regulation and has been intensely studied. Modification of PML disturbs PML NB assembly and alters PML protein stability, localization and interaction partners. Several key regulatory mechanisms for PML are summarized in Tables 1, 2 and Fig. 2.

**Table 1:** Summary of factors and conditions involved in *PML* transcription, translation, alternative splicing and subcellular distribution

Type of regulation	Extracellular stimuli	Cellular regulators	PML regulation	Refs.
Transcription				
	IFNs, TNFα	Stat1 and Stat2/IRF3/IRF8	Upregulation of *PML* mRNA	[29–31, 109]
	Oncogenic stress	RAS/p53	Upregulation of *PML* mRNA	[32, 33]
	Cytokine or hormone	Stat3/Stat6	Downregulation of *PML* transcription	[34]
Post-transcription				
Alternative splicing			Expression of different PML isoforms with distinct function	[15, 20]
Alternative splicing	Herpes simplex virus-1 infection		Increase in cytoplasmic PML in response to viral infection	[27]
mRNA stability	MicroRNAs delivered by colon cancer cell-derived microvesicles	miR-1246	Targeting *PML 3’*-*UTR* for degradation	[35]
Translation				
	Oncogenic stress	RAS/mTOR RAS/eIF4E	Upregulation of *Pml* mRNA translation *5*′-*UTR in* MEFs	[36]
	TNFα	p38/MNK1	Upregulation of *PML* mRNA translation via *IRES*	[37]
Cytoplasmic PML regulation				
	TGFβ	TGFR	Smad2/3 and SARA-mediated TGFβ signaling	[8]
	Sulforaphane (SFN)		Increases in cytoplasmic PML proportion and nuclear NRF2 accumulation	[28]
	PML 1272delAG and IVS3-1 G mutations		Increases in cytoplasmic PML proportion and inhibition of p53-mediated cell apoptosis	[26]
NB formation				
	Androgen		Decreases in PML NB formation	[160]
	Ionizing radiation		Increases in PML NB formation	[161]
	Cisplatin		Increases in PML NB formation	[161]
	SFN		Decreases in PML NB formation	[28]

The abundance/activity of PML protein can be regulated at the level of transcription, alternative splicing, translation and subcellular distribution. The types of regulation are listed in the first column; the extracellular agent or stress that contributes to the PML regulation is summarized in the second column; regulation factors that target or modify PML are shown in the third column and the final column describes effects of these regulatory factors on PML regulation

**Table 2:** Summary of signaling involved in PML post-translational modification

Type of post-translational modification	Extracellular stimuli	Cellular factors	PML regulation	Refs.
Sumoylation (site)				
K65/K160/K490	ND	RanBP2 /Ubc9	Assembly of PML NBs	[20, 38, 42, 43, 61, 162]
	ND	ZNF451-1	Increases in RNF4-mediated PML degradation	[41]
K65 and K160	As_2_O_3_, Tumorigenic adaptation	PIAS1	Increases in CKII-mediated PML degradation	[44]
ND	Cell cycle	ND	Oscillation of PML sumoylation status	[163]
K65/K160	As_2_O_3_		Sumoylation and sumoylation-mediated ubiquitination and degradation	[51]
ND	TNFα	HDAC7	Upregulation of sumoylation	[45, 84]
ND	Thermal stress/Cellular stress	SENP	Desumoylation/NBs dynamic	[44, 46–50]
K65/K160	Viral infection	LANA2	Upregulation of SUMO2-conjugated sumoylation	[164]
ND	Epstein-Barr virus infection	BZLF1	PML desumoylation and NB breakdown	[124]
ND	Cytomegalovirus infection	IE1	Disruption of PML NBs	[126]
Phosphorylation (site)				
ND	DNA damage	ATR	Nucleolar localization	[6, 52, 67]
S565	Osmotic stress/Cellular stress	CKII	PML degradation	[73]
S518	Hypoxia	CDK1/2	Increases in KLHL20-meidated PML ubiquitination and degradation	[58]
ND	Cell cycle	Aurora kinase A	PML hyper-phosphorylation	[72]
S403 and S505	EGF, oncogenic adaptation	ERK2	Increases in Pin1-mediated PML degradation	[70, 71]
S527 and S530	As_2_O_3_	ERK1/2	Increases in PML sumoylation and PML-mediated apoptosis	[69]
S117	γ-irradiation	Chk2	Increases in PML-mediated Apoptosis	[66]
S8, S36, and S38	DNA damage	HIPK2	Increases in PML-mediated Apoptosis	[68]
S403 and T409	Mitogenic stimuli	BMK1/ERK5	Inhibition of PML-mediated p21 suppression for cancer cell proliferation	[165]
S518	ND	SCP1/SCP3	Blockade of CDK1/2-Pin1-KLHL20-PML regulatory loop and PML-mediated anti-angiogenesis	[153]
Ubiquitination				
	As_2_O_3_	E6AP	PML degradation	[55, 56]
	ND	SIAH1 and SIAH2	PML degradation	[57]
	ND	UHRF1	PML degradation	[166]
	Hypoxia	KLHL20	PML degradation	[58]
	As_2_O_3_	RNF4	Catalyzing sumoylation-dependent degradation, increase in PML NB formation	[51, 53, 54]
	HSV-1 infection	ICP0	PML degradation	[125]
	As_2_O_3_	RNF111 (Arkadia)	Catalyzing sumoylation-dependent degradation	[59]
Isgylation				
	Retinoic acid	UBE1L/USP18	PML-RAR degradation	[62, 63, 167]
Acetylation (site)				
K487 and K515	ND	p300	Increases in PML sumoylation	[74]
K487	H_2_O_2_	Sirt1/Sirt5	Deacetylation of PML, increase in K490 sumoylation	[75, 77]
K487	ND	Sirt1	Promotion of PML/PER2-induced BMAL1/CLOCK transcriptional activity	[76]
Protein level regulation				
	H_2_O_2_	Pin1	Decreases in Pin1-PML association and Pin1-mediated PML degradation	[71]
	IGF-1, hypoxia	Pin1	Increases in Pin1-PML association and Pin1-mediated PML degradation	[108]

Post-translational modification of PML controls multiple PML properties, such as protein-protein interaction, stability, NB formation and its ability to regulate transcription and apoptosis. The types of PML post-translational modification and modification sites are listed in the first column; the extracellular agent or stress that contributes to the PML post-translational modification is summarized in the second column; regulation factors that target or modify PML are shown in the third column and the final column describes effects of these regulatory factors on PML post-translational modification

*ND* Not determined

### Transcriptional and translational control of PML

Transcriptional regulation of PML is a critical mechanism for cells to respond to environmental changes. All interferons (IFNs) have been shown to induce *PML* mRNA and protein levels and consequently increase the size and number of PML NBs [29]. Transcriptional up-regulation of *PML* mRNA by IFNs is mainly mediated by the IFN downstream transcription factors, signal transducers and activators of transcription (Stats) and their cis-elements in the *PML* promoter, including IFN-stimulated response elements (ISRE; -GAGAATCGAAACT-) and gamma-activated sites (GAS; -TTTACCGTAAG-) [30]. These IFN responsive cis-elements are activated by both type I and type II IFNs. However, deletion of the GAS element from the promoter only attenuates cellular response to type II IFNs [30]. Also, the interferon-induced regulatory factors, IRF3 and IRF8, are found to bind either ISRE or GAS element and activate *PML* transcription [31]. In addition to promoter-driven transcriptional regulation, p53 can bind the *PML* coding region and when overexpressed, up-regulates *PML* transcription [32]. Through a similar mechanism, RAS-induced p53 up-regulates PML and subsequently promotes cellular oncogenic senescence [33]. Conversely, Stat3 and Stat6 suppress *PML* expression during mammary gland development [34].

PML expression is also subjected to regulation by several post-transcriptional mechanisms. Alternative splicing of *PML* primary transcripts gives rise to multiple *PML* isoforms, all of which possess unique *3*′-*UTRs* (Fig. 1b). A recent finding suggests that *mir*-*1246* targets *PML I* mRNA and reduces its accumulation [35]. In contrast, overexpressed *K*-*RAS* enhances translation of *PML* mRNA in a manner that is mTOR/eIF4E- and *PML 5*′-*UTR*-dependent [36]. During our investigation on the mechanism by which TNFα induces PML protein levels, we identified a well-conserved, 100-nt internal ribosome entry site (IRES) upstream of the translation initiation site. This IRES is further activated by the p38-MNK1 axis to increase PML protein synthesis [37].

### Post-translational modification and subsequent effects on PML protein turnover, ubiquitination (Ub) and Ub-like protein modification

PML protein is covalently conjugated to small protein modifiers, including ubiquitin, SUMO, and possibly ISG15. These modifications require distinct E1/E2/E3 ligation systems. Three lysine residues, K65, K160 and K490, have been identified as the major sumoylation sites [38]. Four SUMO family proteins, including SUMO1, SUMO2, SUMO3 and SUMO5 are known to modify PML at different lysines and sumoylation at different lysine residues has distinct functional consequences [38, 39]. Given that the sumoylation of PML is important for protein-protein interactions, a mechanism underlying sumoylation-dependent biogenesis of PML NBs has been proposed [7, 40]. However, mutations at the three major sumoylation sites reduce but do not completely abolish PML NB formation [24]. Three SUMO E3 ligases, RanBP2, PIAS1 and ZNF451-1 have been suggested to promote PML sumoylation at K490 and K65/K160, respectively [41–44]. Additionally, HDAC7 harbors sumoylation E3 ligase-like activity and promotes PML sumoylation by a HDAC activity-independent manner [45]. Interestingly, loss of *RanBP2* and *HDAC7* significantly reduces the size and number of PML NBs, while knockdown of *PIAS1* increases PML NBs [42–45]. Several pathways or factors have been found to promote PML desumoylation including the SUMO-specific protease (SENP) family. Of this family, all of the SENPs, except SENP4, are capable of removing SUMO conjugation from PML, thus contributing to the dynamic change of PML NBs [46–50]. DNA damage agents and oxidative stress inducers such as arsenic trioxide (As_2_O_3_) also regulate PML sumoylation and PML NB maturation [6, 51, 52]. As_2_O_3_ has been reported to promote NB formation by directly oxidizing PML cysteine residues and by inducing intracellular ROS production, thus facilitating PML sumoylation and degradation [53, 54].

PML protein abundance is regulated by Ub-mediated turnover through multiple mechanisms. Several E3 ligases have been identified that promote PML poly-ubiquitination. As a PML *bona fide* ubiquitin E3 ligase, E6AP interacts with PML and overexpression of E6AP promotes PML poly-ubiquitination and degradation [55]. Partial depletion of E6AP in lymphoid cells results in an accumulation of PML protein and thereby enhances cell susceptibility to genotoxic stress-induced cell death [55, 56]. Similarly, RING-finger ubiquitin E3 ligases, SIAH1 and SIAH2, interact with the PML RING domain and overexpression of these two proteins results in PML and PML–RARα degradation in a proteasome-dependent manner [57]. Under hypoxic conditions, the hypoxia transcription factor HIF1α upregulates KLHL20, an E3 subunit, which promotes degradation of PML in a CDK2- and Pin1-dependent manner, thus promoting prostate cancer progression [58]. Notably, the RNF E3 ubiquitin ligase family in mammalian cells, including RNF4 and RNF111, contains a SUMO interacting motif [59]. In As_2_O_3_ treated APL cells, the SUMO ligase PIAS1 promotes PML sumoylation and facilitates the recruitment of RNF4/RNF111 to PML for poly-ubiquitination and degradation [59, 60]. Through the C-terminal SIM, PML also can interact with sumoylated proteins, including PML itself [20, 38, 61].

It has also been suggested that PML can be modified by ISG15 conjugation (Isgylation). This SUMO-like small protein is induced by type I interferons, lipopolysaccharide or viral infection. Retinoic acid (RA) induced expression of the E1-like ubiquitin-activating enzyme (UBE1L), which enhances ISG15 conjugation of the PML domain of PML/RARα, causing its degradation [62, 63]. Consistently, down-regulation of the ISG15 deconjugating enzyme, USP18, results in an increased fraction of isgylated PML–RARα which leads to a decrease in PML–RARα protein levels, and subsequent APL cell apoptosis [64]. In summary, isgylation of PML may contribute to the PML degradation process.

### Phosphorylation

A major function of PML phosphorylation is to regulate PML protein turnover and PML NB formation, in response to extracellular stimuli such as growth factors and stress conditions such as mitogens, DNA damage and oncogenic stress [65]. When cells are confronted with DNA damage, UV exposure or double stand breaks, PML NBs initially go into a forced fission state because of topological changes of chromatin and hence increase the overall number of small NBs, called PML microbodies [52]. In the late phase of DNA damage repair (DDR), these PML microbodies are thought to assist in the repair process and their propagation is regulated by the DNA checkpoint kinases, Chk2, ATM and ATR. Thus, PML appears to be a sensor and mediator of DNA damage [52]. Furthermore, in response to γ-irradiation, Chk2 phosphorylates PML at S117, thus promoting apoptosis [66]. During doxorubicin induced DNA damage, PML is phosphorylated by the ataxia telangiectasia Rad-3 related kinase (ATR) and accumulates in nucleoli where it sequesters the p53 ubiquitin ligase MDM2, resulting in p53 stabilization [67]. In Adriamycin-induced DNA damage, HIPK2 induces PML phosphorylation at serine 8 and 38, followed by PML sumoylation, stabilization and ensuing apoptosis [68]. Similarly, several ERK1/2 phosphorylation sites on PML have been identified and ERK2-mediated PML phosphorylation has been shown to increase PML sumoylation in response to As_2_O_3_-induced apoptosis [69]. In addition, S403 and S505 of PML are phosphorylated by ERK2 and are essential for Pin1-induced PML degradation [70, 71]. In response to hypoxia, CDK1/2 phosphorylates PML at S518 and promotes Pin1-mediated, cullin3-KLHL20-dependent PML poly-ubiquitination and degradation in prostate cancer cells [58]. Given that PML protein levels oscillate during the cell cycle, besides CDK1/2, another cell cycle related kinase, Aurora kinase A can also phosphorylate PML at several serine residues, implying that phosphorylation of PML may be involved in cell cycle control [72]. Lastly, cellular stresses, such as DNA damage and osmotic stress, promote CK2-mediated PML phosphorylation, leading to PML poly-ubiquitination and degradation promoting cell survival [73].

### Other modifications

Using the HDAC inhibitor TSA, it was shown that PML is acetylaed at two lysine residues, K87 and K515 by p300 [74]. This acetylation can be removed by the class III deacetylase, Sirt1 [75] and Sirt5. K487 is the major acetylated residue and PML deacetylation by Sirt1 promotes PML-mediated PER2 nuclear localization, thus enhancing PER2-mediated circadian gene expression [76]. Furthermore, high concentrations of hydrogen peroxide (H_2_O_2_), an oxidative stress inducer, promotes PML nuclear localization and sumoylation at K490, doing so by recruiting Sirt1 and Sirt5 to catalyze K487 deacetylation [77]. Reciprocally, a loss of *SIRT1* or *SIRT5* by shRNA knockdown induces K487 acetylation, reduces K490 sumoylation and attenuates H_2_O_2_-induced cell death. Thus, K487 acetylation and K490 sumoylation are mutually exclusive. Such regulation may not be limited to PML.

## Regulation of cellular processes by PML

### Regulation of transcription by PML

PML has Ying-Yang effects on the regulation of transcription, but the underlying mechanism remains unclear. Although Chromatin immunoprecipitation (ChIP)-PCR studies have shown that PML can be recruited to promoter regions [78, 79], it is not clear whether nucleoplasmic PML or PML in NBs associates with chromatin and how PML can regulate transcription negatively and positively. This issue is critically important because PML NBs are cellular sensors and their assembly and disassembly are tightly regulated by redox and nutrients [80]. However, this issue is also difficult to address, in part, because there was no available mutant that distinguishes between PML in the nucleoplasm and in NBs. Several observations support the model in which PML regulates transcription through its ability to sequester transcription factors in PML NBs and block their access to chromatin. For example, many transcription factors including the transcriptional coactivator, histone acetyltransferase CBP and transcriptional corepressor proteins Ski and N-CoR are found to colocalize with PML in NBs, often in a signal-dependent manner [13, 81]. The corepressor Daxx is sequestered by PML NBs from repressing GR [82] and Pax3 [83] target genes in response to As_2_O_3_ treatment. Induction of PML NBs by TNFα can sequester nuclear HDAC7 and derepress HDAC7-mediated gene repression in HUVECs [45, 84]. Under conditions of cellular senescence, the induction of PML NBs by oncogenic RAS (RasV12) redirects RB-E2F complexes into heterochromatic regions and blocks E2F activation, thus facilitating senescent processes [78]. Consistent with the sequestration model, PML NBs contain neither chromatin nor nascent RNAs, suggesting that they are not directly involved in transcription [21]. However, immunohistochemistry and microscopic studies suggest that PML NBs physically associate with chromatin [85, 86]. So far, there is no clear evidence to rule out or conclude that PML NBs may directly involve in transcription regulation.

### Regulation of mRNA translation by PML

PML is capable of sequestering the translation initiator eIF4E in the nucleus, thus blocking nuclear export of eIF4E-associated mRNAs and their subsequent polypeptide synthesis [87, 88]. In cap-dependent protein synthesis, the translation initiator eIF4E binds to the 5′ m^7^G cap of most mRNAs that are poised to be translated in the cytoplasm. However, an estimated 70% of cellular eIF4E is localized in the nucleus and this observation intuitively raises a question of whether eIF4E has other functions [89]. Indeed, through binding to structurally conserved *3*′-*UTR* elements in some capped mRNAs, including *cyclin D1* and *Pim1*, nuclear eIF4E facilitates export of these mRNAs to the cytoplasm [87]. The interaction between PML and eIF4E in the nucleus attenuates eIF4E binding to eIF4E-sensitive mRNAs and blocks their nuclear export, thereby lowering protein synthesis of these mRNAs [87]. Furthermore, other translation factors, such as eIF3 and eEF1A1, have been reported to interact with PML. Despite the biochemical interaction of PML with multiple translation factors, it is still unclear whether PML directly regulates ribosome loading or participates in the peptide synthesis [90].

### Regulation of protein post-translational modifications and protein-protein interactions by PML

Since PML associates with several protein modification enzymes, the dynamics of PML NBs have been shown to play a role in protein phosphorylation, sumoylation, ubiquitination and acetylation [13]. HIPK2, p53 and CBP are recruited to PML NBs in response to ultraviolet radiation and this recruitment facilitates HIPK2-mediated p53 phosphorylation and ensuing p53 acetylation by CBP and subsequent p53-mediated apoptosis [91, 92]. The acetylation and deacetylation of p53 is dynamically regulated in PML NBs. Sirt1 serves as a deacetylase to balance p53 acetylation. Overexpression of Sirt1 promotes p53 de-acetylation, which represses p53-mediated transactivation, consequently antagonizing PML-mediated cell senescence [93].

PML also enhances dephosphorylation of a subset of proteins. For example, loss of *Pml* in mouse neural progenitor cells results in mis-localization of protein phosphatases 1A (PP1A), 2A (PP2A) and RB, which leads to RB hyper-phosphorylation, cell proliferation and dysregulation of neuronal development [94]. Loss of *Pml* also blocks PP2A-mediated nuclear AKT dephosphorylation, enhances AKT activity and exacerbates AKT-induced tumorigenesis in *Pten*^+/−^, *Pml*^−/−^ mice [4]. Upon genotoxic stress, IKKε translocates to the nucleus and is recruited to PML NBs, where IKKε is sumoylated by PML-associated SUMO E3 ligase, TOPORS and the sumoylated IKKε is capable of phosphorylating NFκB subunit p65 and contributes to the anti-apoptotic function of NFκB [95].

Some PML-associated protein-protein interactions are sumoylation-dependent through its C-terminal SIM [20, 40, 96, 97]. Through this interaction, several sumoylated proteins can be recruited to PML NBs. For example, As_2_O_3_ induces sumoylation of the antioxidant response transcription factor, NRF2, recruitment to the PML NBs and degradation by RNF4-mediated proteolysis [98]. Furthermore, under osmotic stress, CK2-induced Daxx sumoylation enhances its association with PML and promotes Daxx-mediated anti-apoptotic gene repression [97]. Noticeably, in addition to serving as a platform for SUMO conjugation and protein-protein interactions, PML also directly sumoylates its substrates, such as p53, MDM2 and nuclear misfolded proteins [99, 100]. Chu and Yang have characterized at least seven members of TRIM family proteins, including PML, as potential SUMO E3 ligases which promote MDM2 sumoylation in vivo [99]. Furthermore, the in vitro sumoylation of MDM2 and p53 using recombinant PML protein demonstrates PML as a *bona fide* SUMO E3 ligase [99]. The structural integrity of conserved RING and B-box domains in PML appears required for a full SUMO ligase activity [99]. Since several TRIM proteins also possess ubiquitin E3 ligase activities and PML is highly associated with this conjugation, it will be interesting to investigate whether PML, like TRIM 27 functions as a dual SUMO and ubiquitin E3 ligase [99]. These findings highlight a potential important physiological and pathological function of PML SUMO ligase activity [100].

Several ubiquitin E3 ligases and deubiquitin enzymes have been found in PML NBs. In response to INFα and γ stimulation, an increase in the number of PML NBs results in sequestration of the ubiquitin E3 subunit KLHL20 to NBs. This results in dissociation of KLHL20 from its substrate DNAPK and further enhancing DNAPK stabilization and DNAPK-mediated apoptosis and autophagy [101]. Additionally, upon doxorubicin-induced DNA damage, PML blocks p53 poly-ubiquitination and degradation by sequestering the p53 ubiquitin E3 ligase, MDM2, to PML NBs [67]. Moreover, PML compromises de-ubiquitin enzyme HAUSP/USP7-mediated PTEN deubiquitination by antagonizing Daxx, a positive regulator of HAUSP activity. Since PTEN mono-ubiquitination is a prerequisite for its nuclear retention and tumor suppressor functions [102], *Pml* knockout MEFs or PML dysfunction in APL cells results in PTEN de-ubiquitination and nuclear exclusion [102].

### Other cellular function of PML

Through its association with the DDR kinases, ATR and HIPK2, PML is a regulator of DNA repair processes [52, 66–68, 91, 92]. In addition to PML’s nuclear function, recent findings demonstrated that cytoplasmic PML can regulate MAMs calcium release to mitochondria and consequently controls autophagy and apoptosis [9, 10]. Also, cytoplasmic PML is involved in TGFβ signal activation [8]. Treatment with the antioxidant SFN in HUVECs reduces nuclear PML, thereby promoting ROS levels, NRF2 activation [28] and induction of antioxidant gene expression. As a cellular redox sensor, PML is a major regulator for redox homeostasis. In *Pml*^−/−^ MEFs and mouse liver, the PML deficiency leads to a loss in essential proteins for mitochondrial complex II function, including *Sdha*-*d, Sdhaf1* and *Sdhaf2* and results in the dysfunction of mitochondria and ROS elevation [28]. These data suggest that PML is a key player capable of rebalancing cellular oxidoreduction status in response to different redox perturbations.

## Physiological and pathological role of PML

### PML plays a role in tumor suppression

The tumor suppressor activity of PML was clearly demonstrated based on the higher incidence of tumor formation in *Pml* knockout mouse models [5]. In the context of *Pml* deficiency, additional oncogenic mutations or tumor suppressor depletion significantly exacerbates tumor formation [4, 73]. Consistent with the mouse studies, low PML expression is observed in several types of human cancers, further supporting PML as a guardian to prevent tumorigenesis [12, 103, 104]. In the past decades, a body of in vivo and in vitro studies have elucidated PML’s function in the blockade of cell growth, cell migration and angiogenesis, thus strengthening a role of PML in the tumor suppression. The major findings are summarized as follows: (1) PML stabilizes the tumor suppressor protein p 53 resulting in a positive feedforward loop to promote cell apoptosis and senescence [32, 105]; (2) PML also promotes p53-independent apoptotic pathways, including calcium release from ER and the induction of pro-apoptotic gene expression through sequestration of the transcription repressor, Daxx [10, 106]; (3) PML is transcriptionally and translationally up-regulated by oncogenic K-RAS and is required for K-RAS-induced cellular senescence [33, 36]; (4) PML is regulated by DNA-damage-responsive kinases ATR/Chk2 and is required for DNA damage-induced apoptosis [67]; (5) PML associates with telomeric DNA and telomerase, resulting in telomere instability-induced cell senescence [107]; (6) PML has the potential to suppress breast cancer cell and endothelial cell migration [37, 71, 108] and (7) PML negatively regulates tumor-associated angiogenesis as discussed later [35, 109, 110].

### The role of PML in the maintenance of hematopoietic stem cells and drug-resistance

Recent studies proposed that PML is implicated in the maintenance of cancer stem cells and in the promotion of anti-cancer drug resistance [111]. In glioma, treatment with therapeutic kinase inhibitor against PI3K, AKT or mTOR increased PML protein levels, which may contribute to drug resistance [112]. Therefore, targeting PML could be an approach to reduce the population of drug-resistant cancer cells. Indeed, knockdown of *PML* or treatment of glioblastoma cells with the PML degradation agent, arsenic trioxide, inhibited cancer stem cell growth as well as glioblastoma tumor growth in a mouse xenograft model [113]. The therapeutic potential of As_2_O_3_ on glioma is partially attributed to PML degradation which consequently leads to the PML-associated C-Myc degradation [113]. Because C-Myc is preferentially expressed in cancer stem cells, treatment of PML-degradable agents is limited to the cancer stem cell population and not to matched non-stem cancer cells. In chronic myelogenous leukemia (CML) patients, PML expression levels are negatively correlated with prognosis outcomes. Utilizing a classical p210^BCR-ABL^ mouse leukemia model, Ito et al. showed that depletion of *Pml* in p210^BCR-ABL^-positive leukemic cells was unable to establish CML-like phenotype after serial bone marrow transplantation [114]. PML does so by up-regulating PPARδ, a master transcription factor that regulates fatty acid metabolism by modulating fatty acid oxidation gene expression. Thus, it was concluded that the PML-PPARδ-Fatty acid oxidation (FAO) axis promotes asymmetric division of HSC, a key step keeping stem cell self-renewal from HSC exhaustion [114]. PML also plays a key role in maintaining progenitor cell quiescence and preserving progenitor cell pluripotency, thus contributing to normal mammary gland and neuron development [34, 94]. Also, a PML-PGC1-PPARα-mediated FAO increase was recapitulated in breast cancer stem cells. It was hypothesized that the breast cancer stem cells escape apoptosis and use ATP produced by FAO as an energy resource for survival [115]. Together, these observations suggest that PML is a key regulator of self-renewal for some types of stem cells and stem-like cancer cells [113–115].

### PML function in physiological metabolism

In a recent study, Ohsaki and Fujimoto et al. identified a novel function of nuclear PML in the biogenesis of nuclear lipid droplets which presumably play a key role in the formation of the nucleoplasmic reticulum and nuclear lipid metabolism [116]. Using human biopsy materials and an obese mouse model, Carracedo and Pandolfi et al. found that hepatic PML protein levels are increased in obese subjects which strongly correlated with liver steatosis, suggesting a role for PML in hepatic function in response to obese conditions [117]. Indeed, an earlier study by Cheng and Kao demonstrated that the depletion of *Pml* in mice decreases liver fatty acid accumulation after long-term Western diet feeding and in turn protects mouse liver from dysplastic nodules [118]. Dysplastic nodules are histologically visible benign lesions which follow liver steatosis and are viewed as an early stage of cirrhosis and hepatocellular carcinoma (HCC). It is still unknown how PML contributes to this process while in 50% HCC patients, PML protein levels are elevated compared to non-neoplastic liver tissue [119]. Since PML controls FAO in stem cells, it is possible that PML participates in the liver disorder by interfering the liver metabolism [114]. Analyses of the microarray data in *PML*-depleted HUVECs suggest that PML is involved in the regulation of a large cohort of metabolic gene expression [115, 120]. Along the same line, loss of *Pml* in mice re-patterns glucose and fatty acid metabolism gene expression in metabolically active tissues, such as muscle and liver, increases metabolic rate and resists obese symptoms induced by a Western diet [118]. It is worthy to note that different results in PML metabolic function and obese predisposition have also been reported [115, 121]. Differences in mouse strains, diets, aging and microbial environment may contribute to the inconsistent phenotypes [115, 118, 121]. Also, since PML participates in brain function, the changes in behavior and appetite after *Pml* depletion likely contributes to eating disorders and final experimental outcomes [118].

Similar to that found in *Pml* KO mouse muscle and liver [118], elevated AMPK phosphorylation was also repeatedly observed in *Pml*^−/−^ MEFs, which in turn promotes sustained autophagy through the AMPK-mTOR-Ulk1 pathway [9]. Autophagy is a cell survival mechanism that initiates membrane bound autophagosome formation, facilitates cytosolic protein recycling and saves energy usage under stressful conditions. By this means, *Pml* knockout MEFs are able to survive under condition of nutrient deprivation but are more sensitive than the wild-type cells to autophagy inhibition [9]. Thus, PML can function as a nutrient sensor that maintains metabolic homeostasis.

In addition to regulation of cytoplasmic protein catabolism via autophagy, PML also controls nuclear protein degradation through the SUMO-Ubiquitin ligation system. In a recent study, Guo et al. found that PML preferentially targets misfolded nuclear proteins and functions as a *bona fide* SUMO E3 ligase to promote SUMO2/3-dependent poly-sumoylation [100]. In the absence of *Pml*, the nuclear ubiquitin E3 ligase, RNF4 is unable to ubiquitinate misfolded proteins destined for proteasomal degradation and thus results in the accumulation of misfolded proteins in the nucleus. For examples, PML-mediated degradation plays a key role in the clearance of neurodegeneration-associated polyglutamine (polyQ) mutant proteins, Atxn1 82Q and Htt 97QP, as well as overexpressed, aggregate-prone TDP-43 in the nucleus [100]. This is further supported by the observation that loss of *Pml* exacerbates neurodegeneration phenotypes in a mouse model expressing Atxn1 82Q [100].

### Anti-viral PML functions

Viral infection is viewed as a stress condition in mammalian cells. PML NBs are disrupted upon viral infection and PML expression is induced in response to inflammation, suggesting its role in the anti-viral defense system [122]. An earlier study suggested that the nuclear-replicating DNA viruses preferentially target PML NB [123]. Upon herpes simplex virus 1 (HSV-1) infection, the viral regulatory protein, ICP0, colocalizes with PML and promotes PML degradation and disassembly of PML NBs [123]. Additionally, the expression of the immediate-early protein BZLF1, induced by Epstein-Barr viral infection, competes PML for sumoylation and consequently leads to PML NB disruption [124]. A similar scenario is also recapitulated by the viral IE1 protein during Cytomegalovirus infection [125, 126]. One major function of PML against viral infection is to sequester viral core components required for viral replication. For example, PML IV has been shown to interact with 3D polymerase of the RNA virus, encephalomyocarditis virus (EMCV) and sequester it to PML NBs, therefore blocking virus propagation [127]. Alternatively, HIV pre-integration complexes induce nuclear PML shuttling to cytoplasm, in which PML sequesters the HIV genome and blocks the viral transduction in the early infection stage [128]. Most importantly, *Pml*^−/−^ MEFs are more sensitive to rabies viral infection and *Pml* KO mice are susceptible to lymphocytic choriomeningitis and vesicular stomatitis viral infection [129]. In addition to viral infection-induced acute responses, the infection of hepatitis virus and papillomavirus also results in liver and cervical cancers [130]. Since PML has dual function in the defense of viral infection and tumor suppression, it has been proposed that PML participates in the virus-induced tumorigenesis. Taking hepatitis C virus (HCV) viral infection for example, HCV core protein can inhibit p53 tumor suppression function by targeting PML [131]. HCV transgenic mice are susceptible to liver tumor under *Pml* null background, further supporting an idea that PML blocks HCV-mediated tumorigenesis [132]. Although most studies demonstrated PML’s function against viruses, other reports suggested that nuclear PML may contribute to viral proliferation in some cases [133].

### PML function during the inflammatory response

In addition to its role in anti-bacteria or -viral infection, PML also participates in innate and adaptive immunity. The detrimental granulomatous lesion, botryomycosis, has been observed when bacterial infection occurred in *Pml*^−/−^ mice due to impaired macrophage function [134], suggesting that PML is a regulator of inmate immunity. Indeed, when challenged by bacterial LPS, *Pml*^−/−^ mice showed reduced septic shock and acute hepatitis, partially due to a decreased response to TLR-mediated NFκB survival signaling. Moreover, bacterially infected *Pml*^−/−^ MEFs are incapable of producing IL6, a key cytokine critical for the acute inflammatory response [134]. Similarly, IL-1β production is largely reduced in *Pml*^−/−^ macrophage due to impaired NLRP3 inflammasome assembly, a key step in the process of IL-1β maturation [135]. The activation of the NLRP3 inflammasome requires PML and is presumably induced by cytoplasmic PML [135]. At the onset of an acute inflammation episode in response to viral infection, TNF and IFN are two early cytokines released into the microenvironment. Interestingly, both cytokines are key PML inducers [29–31, 109]. Reciprocally, PML is required for IFN-mediated anti-viral activity that includes activation of Stats, induction of Stat-associated interferon-stimulated genes (ISGs) and promotion of IFN-induced cell apoptosis [110, 127, 136, 137]. Knockdown of *PML* reduces Stat1 function in activating target gene expression by interfering with Stat1 phosphorylation and DNA binding [137]. Knockout or knockdown of *PML* does not affect IFNα-induced transient Stat1 phosphorylation or nuclear translocation; however, IFNα-induced Stat1 and Stat2 isgylation are reduced [110]. This observation is accompanied by reduced expression of a subset of Stat1-activated genes. This implies a role for PML in the regulation of Stat1 function [110]. Previous studies suggested that the regulation of Stat1 by PML mainly resulted from nuclear PML [137], however, whether nuclear or cytoplasmic PML regulates Stat1-isgylation remains unclear and warrants further investigation [137]. In addition, in response to IFN-γ stimuli, PML associates with and protects the major histocompatibility complex class II (MHCII) transactivator (CTIIA) from degradation, thus facilitating MHC II gene transcription [138]. Notably, PML also promotes IFNβ synthesis by enhancing IRF3-induced *IFNβ* expression. To do so, PML IV sequesters Pin1 and thereby blocks Pin1-mediated IRF3 ubiquitination and degradation [139]. A recent study further elucidated the connection between viral infection and PML-mediated IFN stimulated gene regulation. Upon Human Cytomegalovirus (HCMV) infection, the HCMV IE1 protein associates with PML and inhibits PML-bound Stat1 and Stat2 activity, resulting in low ISG expression and reduced innate immunity [140]. IFNα has been clinically used for liver cancer therapy due to its anti-angiogenic and pro-apoptotic function. This may rely on PML-mediated apoptotic gene activation. Indeed, PML has been shown to elevate IFN-induced apoptosis by up-regulating the Death receptor, TRAIL [141].

In addition to IFN-mediated responses, knockdown of *PML* in TNFα treated HUVECs alters the expression of a large cohort of apoptotic, inflammatory and angiogenic genes [120]. This result suggests a role for PML in TNF-mediated responses. Indeed, PML is induced by TNFα at both the transcription and translation levels [37, 109]. However, how PML works in TNFα-mediated inflammatory response is not completely understood. TNFα was first identified as a tumor cytotoxic agent that promotes cancer cell apoptosis [142]. PML is also involved in p53- and Daxx-dependent apoptosis in response to γ-irradiation, IFN, Fas and TNFα [5, 11, 32, 97, 143]. Several mechanisms have been proposed to elucidate how PML contributes to TNFα-induced cell death. It was first shown that ectopic expression of PML converts cancer cells from TNF-resistant to TNFα-sensitive cell death, in part by sequestering the pro-survival factor NFκB to PML NBs. This is consistent with the observation that expression of cell survival genes, including *TNFAIP3* (A20) and *BIRC5* (survivin) was reduced [143, 144]. Secondly, the FAS-interacting protein, Daxx, interacts with PML. It has been proposed that TNFα/FAS ligand-induced PML NBs suppress Daxx anti-apoptotic target gene expression and promote Daxx’s function in DSIC-mediated apoptosis [11, 106]. Because Daxx binds sumoylated PML, this PML function also requires SUMO modification of Daxx [61]. Finally, PML interacts with and regulates other apoptosis-associated transcriptional co-regulators, including HDACs. Through this mechanism, PML NBs induced by TNFα may contribute to TNFα-mediated inflammatory responses and apoptosis; however, this conclusion requires further investigation [37, 45, 84].

### PML function in neurogenesis and brain cognition function

The histological distribution of Pml protein in mouse brain, including cerebellum, cortex, hippocampus and the brain stem, suggests that Pml may function in regulating neural development and activity [12, 145, 146]. Indeed, Regad et al. first demonstrated that Pml is expressed in neural progenitor cells (NPCs) in the ventricular zone and controls their proliferation [94]. PML recruits the tumor suppressor protein RB and phosphatase PP1α in NPC nuclei, resulting in RB hypo-phosphorylation and high activity. Depletion of *Pml* increases RB phosphorylation and inactivation, consequently promoting NPC proliferation. It also disturbs NPC differentiation and the development of neocortex, and consequently causes a small-size brain phenotype [94]. In addition, multiple neuronal activity regulators, such as PER2 and ARC have been demonstrated to be associated with PML in mature neurons. PML physically interacts with PER2 and this is required for PER2 nuclear localization and PER2-mediated circadian clock gene regulation [76]. In *Pml* knockout mice, the expression of key circadian regulators, such as PER1 and PER2 is reduced in the suprachiasmatic nucleus and the interaction of PML-PER2 is disrupted, leading to irregular circadian rhythms [76]. Also, *Pml* knockout mice have fewer whiskers than the wild-type animals, suggesting that these mice are more active [118].

The activity-regulated cytoskeletal protein (Arc) is indispensable for neuronal synaptic plasticity including long-term potentiation (LTP), long-term depression (LTD) and homeostatic scaling [147]. Upon synaptic stimulation, Arc is highly enriched in neuronal nuclei, where it promotes PML NB formation and PML-mediated repression of the activity-regulatory gene, GluA1 [147]. As a result, the Arc-PML-GluA1 axis helps maintain homeostatic of synapse plasticity [147]. In agreement with basic molecular findings, the *Pml* knockout mice showed lower anxiety-related responses and impaired cognitive function as measured by Morris water maze tests and several other behavior assessments [146]. PML was reported to clear the CNS of toxic protein aggregates in neurodegenerative disease models. The polyQ-containing spinocerebellar ataxia 7 and ataxin 1 are toxic proteins to the CNS which result from abnormal expansion of a polyQ tract in their polypeptide chains [100]. These aggregated proteins colocalize with PML NBs and are degraded in a PML- and proteasome-dependent manner [148]. As described earlier, PML catalyzes sumoylation of misfolded proteins and promotes poly-ubiquitination-mediated and proteasome-dependent protein degradation [100]. Based on these findings and the *Pml*^−/−^ mouse model, the function of PML in CNS regulation is well documented; however, a timely and tissue-specific control of *Pml* knockout is key to a full understanding of PML function in neuronal development and mature neuron function. Whether targeting PML is a good therapeutic strategy for the treatment of neurodegenerative diseases awaits for further evaluation.

### PML function in mammary gland development

Lessons from PML studies on hematopoietic stem cell and neuron progenitor maintenance highlights PML’s function in developmental biology [94, 114]. An analogy has been applied to murine mammary gland development since *Pml* knockout results in a reduction of murine ductal lumen size and mammary gland branches [34]. Li et al. found that the *Pml* expression peaks in virgin mammary tissues but decreases during lactation [34]. The *Pml* expression pattern is negatively correlated with the activities of Stat3, Stat5 and Stat6 [34]. Of these Stats, Stat5 has been reported to regulate differentiation of alveolar cells and Stat6 is required for alveolar lineage expansion [149]. In contrast, Stat3 promotes cell death to terminate alveolar development at the end of lactation [150]. The inverse activation pattern of Stat5, Stat6 and Stat3 with *Pml* expression suggested that Stats may transcriptionally suppress *Pml* expression [34]. This is supported by the observation that *Pml* expression is increased in either *Stat3* or *Stat6* knockout mammary gland [34]. Reciprocally, *Pml* depletion attenuates Stat5 and Stat6 activation, indicating an interplay between PML and Stats in the ductal maturation process [149]. Along with stem cell maintenance in hematopoietic and brain systems, loss of *Pml* in mice enhances ERɑ-positive progenitor cloning efficiency, which may lead to the mammary progenitor repopulation and consequent defects in mammary gland maturation [34]. These observations suggest that PML regulates mammary gland morphogenesis and ductal maturation. Also, PML has been proposed to contribute to the tumorigenesis of triple negative and basal-like breast cancers which resemble breast stem/progenitor cells [115, 151]. It will be interesting to investigate whether breast cancer stem cells and mammary progenitors share mutual features.

### PML function in angiogenesis

In tissue microarrays, PML has been found highly expressed in endothelium, suggesting that it may play a role in angiogenesis and vascular biology [12]. In response to hypoxia, a low oxygen condition frequently shown in cardiovascular lesions and tumor microenvironments, it was found that *Pml*^−/−^ mice showed elevated neovascularization [152]. In the tumor microenvironment, the angiogenic system is hijacked by cancer cells and is central for the promotion of tumor growth and metastasis. Under hypoxic conditions, overexpression of PML suppresses mTOR function by sequestering mTOR in the nucleus thereby blocking mTOR-mediated HIF1 protein expression and angiogenesis [152]. Reciprocally, hypoxia-induced HIF1α upregulated Cul3-KLHL20 ubiquitin ligase which promotes PML ubiquitination and degradation after CDK1/2-mediated PML phosphorylation and subsequent isomerization by Pin1 [58]. As such, prostate cancer cells increase tumor-associated angiogenesis for tumor growth. Along this line, members of the protein serine/threonine phosphatase (SCP) family, dephosphorylate PML and thereby block the CDK1/2-Pin1-KLHL20-PML regulatory loop [153]. The frequent deregulation of SCP1 and SCP3 in clear cell renal cell carcinoma and concomitant PML reduction results in mTOR-HIF1 axis activation, thus increasing tumor-associated angiogenesis and tumor burden in Xenograft models [153]. Alternatively, neuroblastoma contain low level of PML I and PML I-induced expression of thrombospondin-2 (TSP2), a potent angiogenic inhibitor [154]. Overexpression of PML I in aggressive neuroblastoma cancer cell lines restored inhibition of tumor-associated angiogenesis [154]. In addition to an intrinsic reduction of PML in cancer cells, it was shown that colorectal cancer cells are capable of reducing PML protein abundance in endothelial cells. Colon cancer cells release microvesicle-capsulated *miR*-*1246* to the recipient endothelial cells and downregulate *PML* expression and protein abundance by targeting *3’*-*UTR* of *PML I mRNA*, thus promoting endothelial cell migration and tube formation [35].

Additionally, a transcriptome analysis using *PML* knockdown HUVEC implied that PML controls a subset of genes participating in the angiogenic process, including genes that affect cell morphological change, migration and adhesion [36, 37, 109, 110]. Among these PML targeting genes, *ITGB1* mRNA and protein levels are up-regulated in *PML* knockdown HUVECs and MDA-231 breast cancer cells [108, 109]. Thus, upregulation of PML represses *ITGB1* expression and inhibits HUVEC and MDA-231 cell migration [108, 155]. Utilizing a similar mechanism, anti-angiogenic cytokines, TNFα and IFNα suppress *ITGB1* expression by transcriptionally promoting Stat1-mediated PML expression and thus block EC migration and vascular network formation [109]. Moreover, TNFα activates *IRES*-driven *PML* mRNA translation and PML-dependent anti-angiogenesis via its downstream kinases p38-MNK1 [37]. The induction of PML by TNFα results in HDAC7 sequestration in PML NBs and relieves HDAC7-mediated repression of the vessel destructive factor and matrix proteinase, MMP-10 [84]. Like TNFα-mediated anti-angiogenesis, PML up-regulation by IFNα reinforces IFNα downstream angiogenic and angiostatic regulatory loops toward being anti-angiogenic. Mechanistically, PML promotes isgylation and activation of nuclear angiostatic transcription factors, Stat1 and Stat2, but antagonizes angiogenic transcription factor, Stat3, through proteasomal-mediated Stat3 degradation [110]. Manipulation of the isgylation system or ablation of *PML* tips the PML-Stats regulatory loop and alters this IFNα-PML-mediated inhibition of angiogenesis in vitro and ex vivo [110]. Despite the isgylation of Stat1/2 presumably attributed to Stat1/2-mediated anti-angiogenesis, how ISG15 conjugation affects Stat1/2 activity in angiogenic gene expression is still unknown. It has been proposed that isgylation of Stat1/2 regulates their stabilities as in the case for p53 and UBC13 [156, 157]. Further studies on identification of isgylated residues and isgylation-defective mutants should provide information on the functional significance of PML-regulated Stat1/Stat2 isgylation. Altogether, PML functions as a negative regulator in angiogenesis. Manipulation of PML is a key mechanism to regulate tumor-associated angiogenesis and IFN and TNF-mediated angiogenic suppression.

## Concluding and remarks

**Fig. 3:**
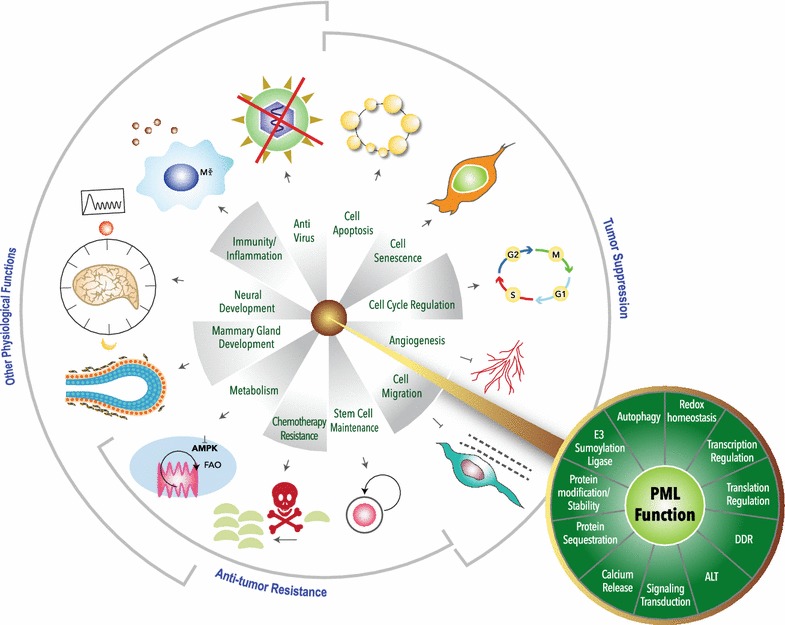
Overview of PML cellular and physiological functions. PML has the ability to form PML NB and interact with more than 170 proteins. Through these interactions, PML has been reported to control various outcomes from gene expression, mRNA translation to protein-level regulation. In a recent study, PML’s enzymatic activity as an E3 sumoylation ligase was validated. Additionally, cytoplasmic PML can regulate calcium transfer, signal transduction and autophagy. All of PML’s known molecular functions are summarized in the green circle. In physiology and pathology, PML was originally found as a tumor suppressor by controlling cell death, cell cycle, angiogenesis and cell migration. In recent studies, PML was also reported as a key player contributing to cancer therapy resistance. Other PML physiological functions in development, immunity and metabolism are also summarized in the grey circle. *ALT* alternative lengthening of telomeres

The main functions of PML are summarized in Fig. 3 and Table 3. The complexity of PML isoforms makes it difficult to clarify and track the precise contribution of each isoform in these processes. Using the CRISPR-knockout technique, experiments with ectopically expressed individual isoforms in a *PML* null background are feasible and could provide insights into the function of individual PML isoform. To date, nuclear PML-mediated tumor suppression function is well-documented both in vivo in mouse models and in many types of human cancer [5, 12, 25]. The cytosolic PML also likely functions as a tumor suppressor by controlling calcium transfer and autophagy while the detailed mechanisms require further investigation [8, 9]. Interestingly, several recent findings highlighted a role of PML in physiologic and pathologic metabolism, especially in FAO and energy usage control [114, 115, 117, 118]. Although this mechanism is required for hematopoietic and cancer stem cell renewal, the changes in body mass and high AMPK activation in metabolic tissue in *Pml* knockout mice suggest a broad spectrum of PML-mediated metabolic regulation [9, 118]. Nevertheless, some inconsistent observations from *Pml* whole knockout mice are prompting us to investigate PML metabolic function in more strictly defined experimental settings [115, 118, 121]. For example, using a single mouse strain capable of conditionally knocking out *Pml* in metabolic tissues such as liver and muscle and maintaining it under defined conditions will likely clarify discrepancies currently noted between different studies [115, 118, 121].

**Table 3:** Overview of PML physiological function and the corresponding phenotypes in *Pml*^−/−^ mouse

Physiological function	*Pml*^−/−^ mouse phenotype	Known key cellular mechanism
Tumor suppression	1. Susceptible to carcinogen/oncogene-induced tumorigenesis [4, 5] 2. Resistant to irradiation-induced death [5]	1. TGFβ→cPML→Smad2/3 and SARA→apoptosis [8] 2. RAS→P53→PML→Cell senescence and apoptosis [33, 105] 3. DDR→PML**-|**MDM2**-|**P53→apoptosis [67] 4. PML**-|**Daxx→HAUSP**-|**PTEN→apoptosis [102] 5. PML**-|**RB-E2F→Cell survival [78] 6. PML→HIPK2→P53→apoptosis [68, 91, 92] CBP 7. PML→PP2A**-|**Akt→Cell survival [4, 10] 8. IFN→PML→TRAIL→apoptosis [141] 9. TNF→PML**-|**NF-κB→A20/survivin expression**-|** apoptosis [143, 144] 10. FAS/Osmotic stress→PML→DAXX**-|**anti-apoptotic gene expression [97]
Hematopoietic stem cell renewal	1. Loss of CML-like phenotype in p210^BCR-ABL^ /*Pml*^−/−^ mouse model [114] 2. Exhaustion and reduced quiescence in *Pml*^−/−^ hematopoietic stem cells [114]	1. PML→PPARδ→fatty acid oxidation→HSC asymmetric division [114]
Neuron function, development and brain cognitive ability	1. Small brain size [94] 2. Defects in cortex development and macroglia differentiation [94] 3. Loss of learning and memory consolidation [147] 4. Lower anxiety-associated response [146] 5. Loss of *Pml* aggravates the neurodegenerative phenotype in SCA1 mouse model [100] 6. Loss of precision and stability in circadian rhythm [76]	1. PML→PP1α→Rb→neural progenitor cell fate regulation [94] 2. Neural activity stimuli→Arc→PML-NB**-|** GluA1→homeostatic plasticity [147] 3. PML E3 SUMO ligase activity→PolyQ-ATXN7/ATXN1-sumoylation→RNF4-mediated ubiquitination and degradation [100] 4. Sirt1→PML→PER2→BMAL1/CLOCK→circadian rhythm [76] 5. As_2_O_3_**-|**PML→c-Myc→glioblastoma stem cell growth [113]
Mammary gland development and triple-negative breast cancer stem cell maintenance	1. Impaired development of mammary ductal and alveolar structure [34] 2. Defects in mammary progenitor cell fate determination [34]	1. Stat3/Stat5/Stat6**-**mediated Pml regulation is required for normal mammary gland development [34] 2. PML→Sirt1→Ac-GPC1α→GPC1α →PPARδ→Fatty acid oxidation gene expression→breast cancer stem cell survival [115]
Metabolism	1. Slim body, higher body temperature and high energy usage rate in a high fat diet [118] 2. Elevated AMPK Activation in liver and muscle in normal chow or Western diet [118] 3. Reduced lipid metabolic gene expression in *Pml*^−/−^ mouse liver; in this study, *Pml*^−/−^ mouse is prone to obese condition^a^ [115] 4. Loss of *Pml* promotes adipogenesis and adipogenic transcription factor activity^a^ [121]	1. PML**-|**AMPK→FAO→metabolism [118] 2. PML→PPARδ→FAO→HSC self-renewal [114] 3. PML→Sirt1→Ac-GPC1α→GPC1α →PPARδ→FAO gene expression→breast cancer stem cell survival [115] 4. p53→PML (MAM localization)**-|**AMPK-mTOR-ULK1→mitochondria calcium transfer→autophagy [9]
Anti-viral infection	1. Susceptible to liver tumor in HCV transgenic mouse [132] 2. *Pml*^−/−^ MEFs are susceptible to viral infection [129]	1. (ICP0, BZLF1 and IE1)**-|**PML (PML NB disassembly)→anti-HSV1 [123–126] 2. PML **-|** 3D polymerase→virus propagation [127] 3. PML cytosolic translocation **-|**HIV viral genome [128]
Innate immunity and inflammatory response	1. Severe granulomatous lesion and botryomycosis in *Pml*^−/−^ mice upon bacterial infection [134] 2. Impaired macrophage function [134, 135, 158] 3. Low acute inflammatory response [134, 135]	1. IFN→PML→STAT1→anti-viral gene and inflammatory gene expression [127, 136, 137] 2. IFNγ→PML→CTIIA→MHC-II expression [138] 3. PML**-|** Pin1**-|**IRF3→IFNβ expression [139] 4. PML→NLRP3 inflammasome assembly→IFNβ maturation [135] 5. HCMV→IE1**-|**PML→STAT1/STAT2→ISGs expression [140] 6. IFN→PML→TRAIL→apoptosis [141] 7. TNF→PML**-|**NF-κB→A20/survivin expression**-|** apoptosis [143, 144] 8. FAS→PML**-|**Daxx**-|**anti-apoptotic gene expression [11, 97, 106]
Angiogenesis	1. Increased neovascularization upon ischemia [152] 2. Compromised IFNα-mediated angiostatic activity ex vivo in *Pml* knockout mouse [110]	1. Hypoxia: PML**-|**mTOR-Rheb→HIF1→angiogenesis [152] 2. Hypoxia→Cul3-KLHL20**-|** PML**-|** HIF1→angiogenesis [58] Cdk1/2 3. Carcinoma-| SCP1/SCP3→PML**-|**angiogenesis [153] 4. Tumor microvesicle→miR1246**-|**PML**-|** angiogenesis [35] 5. TNF→P38→MNK1→PML**-|** angiogenesis [37] 6. IFN→PML→STAT1**-|**angiogenesis [110] STAT3

PML is a versatile protein involved in several physiological functions. Here, we listed most of them, including tumor suppression, stem cell maintenance, neural and mammary development, metabolism, anti-virus, immunity and angiogenesis. We summarized the phenomenal phenotypes shown in *Pml*^−/−^ mouse. Also, we listed key reported mechanisms regarding with each physiological function

*cPML* cytoplasmic PML

^a^*Pml*^*−*/−^ mice show obese phenotype)

Metabolic disorders such as diabetes and cardiovascular disease are associated with chronic inflammation. Polarization of macrophage is a key step in chronic inflammation. Since PML is a sensor for inflammatory cytokine responses and nutrient status, *Pml* knockout macrophage could be employed to investigate whether the deregulation of *PML* in macrophage affects inflammation-induced diabetic symptoms [134, 158]. The reduction of PML-mediated angiostatic effects is hijacked by cancer cells to create a tumor favorable microenvironment [35]. Conversely, both anti-tumor cytokines, IFN and TNF, promote PML-mediated angiostatic activity in endothelium [37, 109, 110]. Several clinical investigations and pre-clinical models have suggested that manipulation of PML may be feasible therapeutic strategy for some diseases. For example, the low levels of PML in proliferative diabetic retinopathy implies that up-regulation of PML might mediate angiostatic effects and ameliorate these symptoms in diabetic patients [159]. Additionally, since PML contributes to hematopoietic cancer stem cell self-renewal and the aggressiveness of triple-negative breast cancer, directly reducing PML protein levels seems to be a promising strategy to treat some types of cancers. Indeed, results from two recent preclinical studies support this hypothesis. They have treated glioma stem cells and triple-negative breast cancer metastasis with agents promoting PML degradation [113, 151]. While intensive studies have been focused on post-translational modification and regulation of PML, more exciting findings will be revealed in the years to come. Moreover, few details are known about the underlying mechanisms of transcriptional regulation by PML and translational control of *PML* mRNA. New findings on these topics will be important and are highly anticipated. It is also worth noting that a combinational therapy of PML modulating agents with other genotoxic agents has demonstrated a better outcome and provide an effective treatment for a clinical application [113, 151]. Therefore, the clarification of PML function in these treatments is key to developing more effective therapies in the future.

## Abbreviations

3KR: PML mutated in K65, K160, and K490
AKT: protein kinase B
AMPK: 5’ adenosine monophosphate-activated protein kinase
APL: acute promyelocytic leukemia
ARC: activity-regulated cytoskeleton-associated protein
As_2_O_3_: arsenic trioxide
ATM: the protein kinase ataxia-telangiectasia mutated
ATR: ataxia telangiectasia Rad-3 related kinase
Atxn1: spinocerebellar ataxia Type 1 protein
BZLF: Epstein-Barr virus replication activator known as Zta
CBP: CREB-binding protein
CDK: cyclin dependent kinase
ChIP: chromatin immunoprecipitation
Chk2: checkpoint kinase 2
CK2: casein kinase 2
CML: chronic myelogenous leukemia
CNS: central nerve system
CTIIA: major histocompatibility complex class II transactivator
Daxx: death-associated protein 6
DDR: DNA damage repair
DNAPK: DNA-dependent protein kinase
eIF4E: eukaryotic translation initiation factor 4E
eEF1A1: elongation factor 1A1
EMCV: encephalomyocarditis virus
ER: endoplasmic reticulum
ERɑ: estrogen receptor α
FAO: fatty acid oxidation
FAS: apoptosis-mediating surface antigen
GAS: gamma-activated sites
GLUA1: glutamate ionotropic receptor AMPA type subunit 1
H2O2: hydrogen peroxide
HAUSP: ubiquitin-specific-processing protease 7
HCC: hepatocellular carcinoma
HCV: hepatitis C virus
HDAC: histone deacetylase
HIF1: hypoxia-inducible factor 1
HIPK2: homeodomain interacting protein kinase 2
HP1: heterochromatin protein 1
HSV-1: herpes simplex virus type 1
Httex1: huntingtin protein
HUVEC: human umbilical vein endothelial cell
ICP0: Human Herpes Virus (HHV) Infected Cell Polypeptide 0
IE1: immediate-early protein1
IFN: interferon
IP3R: inositol triphosphate receptor
IRES: internal ribosome entry site
IRF: interferon regulatory factor
ISG: interferon-stimulated gene
ISRE: interferon-sensitive response element
ITGB1: integrin β1
KLHL: Kelch-like protein
KO: knockout
Kr: Kremer bodies
LPS: lipopolysaccharide
LTD: long-term depression
LTP: long-term potentiation
MAMs: mitochondria-associated membranes
MDM: mouse double minute 2 homolog
MEF: mouse embryonic fibroblast
MHCII: major histocompatibility complex class II
MMP-10: matrix metallopeptidase 10
MNK: mitogen-activated protein kinase interacting protein kinases
mTOR: mechanistic target of rapamycin
Myc: Myelocytomatosis Viral Oncogene Homolog
NBs: nuclear bodies
NES: nuclear export sequence
NF-κB: nuclear factor kappa-light-chain-enhancer of activated B cells
NLRP3: NLR family pyrin domain containing 3
NLS: nuclear localization sequence
NPCs: neural progenitor cells
Nrf2: nuclear factor (erythroid-derived 2)-like 2
PER2: circadian clock protein PERIOD 2
PGC: proliferator-activated receptor gamma coactivator
PIAS1: protein inhibitor of activated STAT 1
PIN1: peptidylprolyl cis/trans isomerase
PolyQ: polyglutamine
PML: promyelocytic leukemia protein
PP2A: protein phosphatase 2A
PPAR: peroxisome proliferator-activated receptor
PTEN: phosphatase and tensin homolog
RA: retinoic acid
RanBP2: RAN binding protein 2
RARα: retinoic acid receptor alpha
RAS: Rat Sarcoma Viral Oncogene Homolog
RB: retinoblastoma protein
RBCC: ring finger/B box/coiled-coil
RNF: ring finger proteins
ROS: reactive oxygen species
SARA: SMAD anchor for receptor activation
SCA1: spinocerebellar ataxia type 1 gene
SCP: small CTD (carboxy-terminal domain, RNA polymerase II, polypeptide A) phosphatase
Sdhaf: succinate dehydrogenase complex assembly factor
SENP: SUMO-specific protease
SIAH: seven in absentia homolog
SIM: SUMO-interacting motif
Sirt1: sirtuin1
Smad: protein similar to mothers against decapentaplegic
STAT: signal transducers and activators of transcription
SUMO: small ubiquitin-like modifier
TGF: transforming growth factor
TLR: toll-like receptor
TNF: tumor necrosis factor
TOPORS: TOP1 binding arginine/serine rich protein
TRAIL: TNF-related apoptosis inducing ligand
TRIM: tripartite motif
TSP2: thrombospondin-2
Ub: ubiquitin
UBC13: ubiquitin-conjugating enzyme13
UBE1: ubiquitin activating enzyme E1
USP: ubiquitin specific peptidase
UTR: untranslated region
ZNF: zinc finger
