# Correction to: PML: Regulation and multifaceted function beyond tumor suppression

**DOI:** 10.1186/s13578-018-0213-7

**Published:** 2018-03-06

**Authors:** Kuo-Sheng Hsu, Hung-Ying Kao

**Affiliations:** 1 Department of Biochemistry, Case Western Reserve University, 10900 Euclid Avenue, Cleveland, OH 44106 USA; 2 The Comprehensive Cancer Center of Case Western Reserve University and University Hospitals of Cleveland, Cleveland, OH 44106 USA; 3 Present Address: Tumor Angiogenesis Section, Mouse Cancer Genetics Program (MCGP), National Cancer Institute (NCI), NIH, Frederick, MD 21702 USA

## Correction to: Cell Biosci (2018) 8:5 10.1186/s13578-018-0204-8

In the publication of this article [1], there is an error in Table 3. The position of the black arrow and the terms CBP, Cdk1/2 and STAT3 of Table 3 were placed incorrectly in the online version while these were placed correctly in the PDF.

The error:

‘ ’

Should instead read:

‘ ’

CBP is placed below HIPK2 and points P53.

The error:

‘ ’

Should instead read:

‘ ’

Cdk2 is placed below Cul3-KLHL20. Hypoxia points Cdk2 and Cdk2 inhibits PML.

The error:

‘ ’

Should instead read:

‘ ’

STAT3 is placed below STAT1. PML inhibits STAT3 and STAT3 points angiogenesis.

This has now been updated in the original article [1].
